# Derivatives of Trimethoxybenzoic Acid and Gallic Acid as Potential Efflux Pump Inhibitors: In Silico and In Vitro Studies

**DOI:** 10.3390/ijms232214468

**Published:** 2022-11-21

**Authors:** Ana Rita Neves, Fernando Durães, Joana Freitas-Silva, Nikoletta Szemerédi, Paulo Martins-da-Costa, Eugénia Pinto, Marta Correia-da-Silva, Gabriella Spengler, Emília Sousa

**Affiliations:** 1 Laboratory of Organic and Pharmaceutical Chemistry, Faculty of Pharmacy, University of Porto, Rua de Jorge Viterbo Ferreira 228, 4050-313 Porto, Portugal; 2 Interdisciplinary Centre of Marine and Environmental Research (CIIMAR), University of Porto, Novo Edifício do Terminal de Cruzeiros do Porto de Leixões, Avenida General Norton de Matos, S/N, 4450-208 Matosinhos, Portugal; 3 ICBAS—Institute of Biomedical Sciences Abel Salazar, University of Porto, Rua de Jorge Viterbo Ferreira 228, 4050-313 Porto, Portugal; 4 Department of Medical Microbiology, Albert Szent-Györgyi Health Center and Albert Szent-Györgyi Medical School, University of Szeged, Semmelweis utca 6, 6725 Szeged, Hungary; 5 Laboratory of Microbiology, Department of Biological Sciences, Faculty of Pharmacy, University of Porto, Rua de Jorge Viterbo Ferreira 228, 4050-313 Porto, Portugal

**Keywords:** trimethoxybenzoic acid, gallic acid, synthesis, antibacterial, efflux pump inhibitors, structure-activity relationship

## Abstract

The overexpression of efflux pumps is one of the strategies used by bacteria to resist antibiotics and could be targeted to circumvent the antibiotic crisis. In this work, a series of trimethoxybenzoic acid derivatives previously described as antifouling compounds was explored for potential antimicrobial activity and efflux pump (EP) inhibition. First, docking studies on the acridine resistance proteins A and B coupled to the outer membrane channel TolC (AcrAB-TolC) efflux system and a homology model of the quinolone resistance protein NorA EP were performed on 11 potential bioactive trimethoxybenzoic acid and gallic acid derivatives. The synthesis of one new trimethoxybenzoic acid derivative (derivative **13**) was accomplished. To investigate the potential of this series of 11 derivatives as antimicrobial agents, and in reverting drug resistance, the minimum inhibitory concentration was determined on several strains (bacteria and fungi), and synergy with antibiotics and EP inhibition were investigated. Derivative **10** showed antibacterial activity against the studied strains, derivatives **5** and **6** showed the ability to inhibit EPs in the *acrA* gene inactivated mutant *Salmonella enterica* serovar Typhimurium SL1344, and **6** also inhibited EPs in *Staphylococcus aureus* 272123. Structure-activity relationships highlighted trimethoxybenzoic acid as important for EP inhibitory activity. Although further studies are necessary, these results show the potential of simple trimethoxybenzoic acid derivatives as a source of feasible EP inhibitors.

## 1. Introduction

After the discovery of penicillin in 1928 by Alexander Fleming, the treatment of infectious diseases suffered an immediate and profound impact [1]. In the same decade, penicillin-resistant bacteria started to emerge, which ignited the search for new antibiotics. Currently, we face the “antibiotic resistance crisis” due to the overuse and/or inappropriate prescription, as well as extensive veterinary and agricultural use of antibiotics [2]. The fact that antibiotic development is no longer considered to be an economically wise investment for the pharmaceutical industry, a decrease in the number of new antibiotics approved has been observed in recent years. In fact, no new antibiotics have been approved by the Food and Drug Administration (FDA) since 2019, and no new chemical classes of antibiotics have been discovered for over three decades. However, with the continuous emergence of antibacterial resistance, the search for new antibiotics to treat resistant bacteria is highly relevant [2,3,4,5].

Efflux pumps (EPs) are membrane structures that are responsible for detoxification pathways by expelling xenobiotics and are present in every cell. In bacteria, they present a multitude of substrates, in which several antibiotics are included, and their overexpression leads to multidrug resistance. Therefore, the search for compounds that can inhibit these structures constitutes an interesting approach to revert antimicrobial resistance. Although no compound with this effect has reached the clinic as an antibiotic adjuvant, advances in the use of bacterial efflux pump inhibitors (EPIs) have been made in recent years [6].

**Figure 1:**
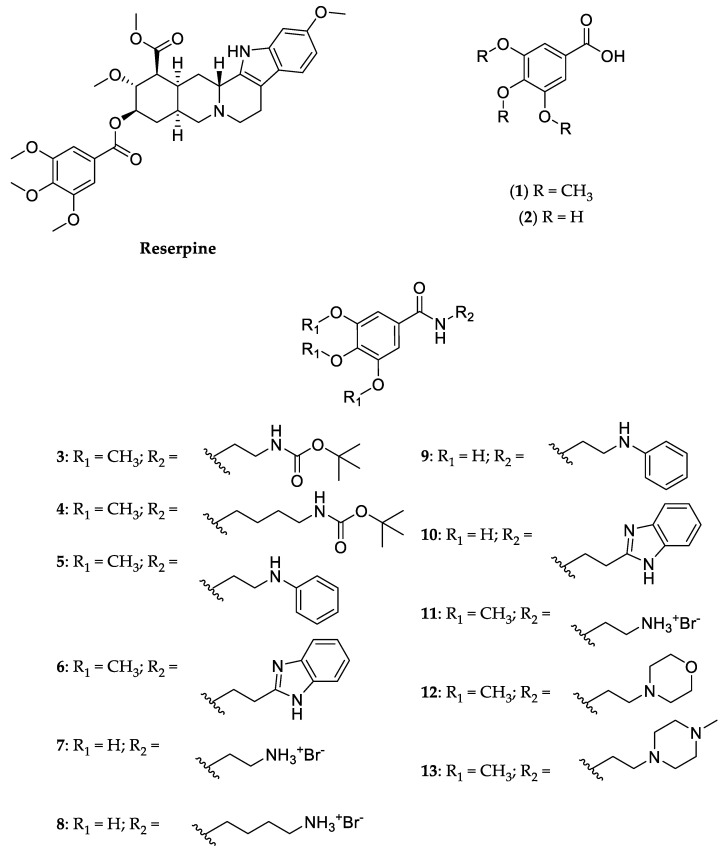
Structure of reserpine, trimethoxybenzoic acid (**1**), gallic acid (**2**) and their derivatives (**3**–**13**).

Our group has recently described the synthesis of “optimized” gallic acid derivatives as antifouling agents [7,8]. Fouling is a phenomenon caused by macro- and microorganisms, such as bacteria and fungi. As part of a screening of compounds with potential antimicrobial/EPI activity, a series of 11 synthetic derivatives of trimethoxybenzoic acid (**1**) and gallic acid (**2**) was studied (**3**–**13**, Figure 1) regarding potential antimicrobial activity and EP inhibition [7,8,9].

Similar to reserpine (Figure 1), a known EPI of the quinolone resistance protein NorA efflux pump, which has also recently been reported as an inhibitor of acridine resistance protein B (AcrB) [10,11], this series includes a trimethoxybenzoic acid moiety in its general structure. However, the use of reserpine, which has been approved as an antihypertensive and antipsychotic, is hindered by its toxicity, which includes hypotension and bradycardia [12,13,14,15]. As such, these derivatives could constitute safer alternatives.

Docking studies were conducted, targeting portions of the AcrAB-TolC bacterial efflux system, from the resistance-nodulation-division (RND) [16], and in a homology model of the NorA efflux pump, from the major facilitator superfamily (MFS) [17]. The obtained results prompted deeper studies for two trimethoxybenzoic acid derivatives (**5** and **6**) and two gallic acid derivatives (**9**, and **10**). The minimum inhibitory concentration (MIC) and synergy with antibiotics were determined for these four derivatives. The derivatives with the best docking scores for bacterial EPs were studied for their potential as EPIs, as well as to other resistance and virulence mechanisms related to EPs, such as influence on biofilm formation and quorum-sensing (QS) inhibition, respectively, and their effect against fungi.

## 2. Results and Discussion

### 2.1. Chemistry

Compound **13** (Figure 1) was synthesized for the first time in this work, while the syntheses of compounds **3**–**11** (Figure 1) was recently published by our group [7]. Compound **12** (Figure 1) was previously reported in a US patent [9].

**Scheme 1:**
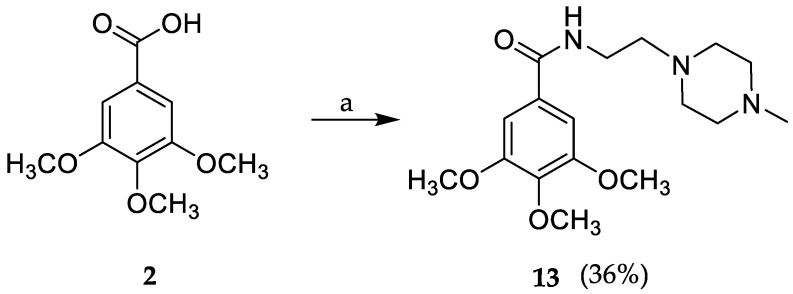
Synthesis of compound **13** through a coupling reaction. a: 2-(4-methyl-piperazin-1-yl) ethylamine, TBTU (2-(1*H*-benzotriazole-1-yl)-1,1,3,3-tetramethylaminium tetrafluoroborate), TEA (triethylamine), THF (tetrahydrofuran), rt (room temperature).

Compound **1** (commercially available at a low cost) was selected as the starting material for the reaction to increase selectivity, since first attempts using gallic acid (**2**) originated several products due to the presence of three phenolic groups (data not shown). Briefly, compound **1** was allowed to react for 15 min with triethylamine (TEA) and 2-(1*H*-benzotriazole-1-yl)-1,1,3,3-tetramethylaminium tetrafluoroborate (TBTU), after which the respective amine was added (Scheme 1). The reaction proceeded at room temperature for 24 h and compound **13** was obtained in 36% yield, after a liquid-liquid extraction, followed by crystallization with ethyl acetate.

The structure of compound **13** was elucidated by ^1^H and ^13^C Nuclear Magnetic Resonance (NMR) and High-Resolution Mass Spectrometry (HRMS, See Supplementary Material, Figures S1–S5).

### 2.2. Molecular Docking

Compounds **1**, **2**, and the library of 11 derivatives **3**–**13** were investigated in silico for their potential as bacterial EPIs using docking studies in relevant EPs present in Gram-positive and Gram-negative bacteria such as the NorA and AcrAB-TolC, respectively. The rationale was to investigate if compounds structurally related to trimethoxybenzoic acid (**2**) that included an amine functional group would have docking scores comparable to those of positive controls. As such, amide derivatives with an amine connected by an aliphatic chain were planned for this study. Due to the simple and feasible procedure, demethylated derivatives were also considered.

Compounds **1**–**13** were subjected to docking studies using AutoDock Vina. Docking studies were performed in the crystal structures of the AcrB (2DRD), AcrA (2F1M), and TolC (1EK9) portions of the AcrAB-TolC efflux system. For AcrB, these studies were performed in sites recently described for reserpine in [11]. Concerning AcrA, two different sites were used: the helical hairpin (HH) and the lipoyl domain (LD) [16]. For TolC, only the lysine residues that interact with the 3,3′-dithiobis(sulfosuccinimidyl propionate) (DTSSP) bifunctional crosslinker [16], were considered.

The NorA pump does not have a crystal structure deposited into the Protein Data Bank (PDB), and a homology model was built. This was achieved using EmrD, an MFS pump present in *Escherichia coli*, whose crystal structure has been deposited in the PDB and is described as the most used model to perform computational studies for NorA (41% similarity and 19% identity) [18,19]. Using Swiss Model, a NorA homology model was generated, and the site used for docking the compounds was the one described for reserpine. The results are shown in Table 1.

From the analysis of the docking results, it can be noted that compounds with the best docking scores for the AcrB portion were compounds **5**, **6**, **9**, and **10**. Compounds **5**, **6**, **9**, and **10** also displayed the best results for the binding core region (BCR) of the NorA homology model.

The results show a better affinity for the AcrB portion of the AcrAB-TolC efflux system, like reserpine. In general, it can be noted that most of the compounds presented the lowest predicted affinity for the AcrA portion, and as such, a model with this part of the pump deleted was chosen for further assays.

Compounds with the best scores (**5**, **6**, **9**, and **10**) were visualized using PyMol, to compare their predicted mode of binding with reserpine, which is presented in the Supplementary material.

### 2.3. Antibacterial Activity and Antibiotic Potentiation

Compounds **3**–**13** were tested against *Escherichia coli* American Type Culture Collection (ATCC) 25922, *Staphylococcus aureus* ATCC 25923, *Pseudomonas aeruginosa* ATCC 27853, *Enterococcus faecalis* ATCC 29212, *Salmonella enterica* serovar Typhimurium SL1344, and the oxacillin- and methicillin-resistant *S. aureus* 272123. Among the tested compounds (**3**–**13**), none was active against the ATCC strains tested. However, compound **10** displayed antibacterial activity against the *S. aureus* 272123 and *S*. Typhimurium strains, of 100 and 50 µM, respectively. All the others did not display an observable MIC at the concentrations tested (MIC > 100 µM). Ciprofloxacin was used as a positive control, with MICs of 1.50 µM for *S. aureus* ATCC 25923 and *P. aeruginosa* ATCC 27853, 3.00 µM for *E. faecalis* ATCC 29212, 0.048 µM for *E. coli* ATCC 25922, 12.5 µM for *S. aureus* 272123, and 6.25 for *S.* Typhimurium SL1344.

For the potentiation of antimicrobials, the bacteria studied were the extended spectrum β-lactamase (ESBL)-producer *E. coli* SA/2 and the vancomycin-resistant *E. faecalis* B3/101. This assay consisted of the determination of the MIC for each antibiotic in the presence of a fixed concentration of each compound. Cefotaxime (CTX) was used against *E. coli* SA/2 and vancomycin (VAN) was used against *E. faecalis* B3/101. The concentration of the compounds was the highest concentration tested in the antibacterial activity assay that did not inhibit the growth of the respective ATCC strain under study (100 µM). MICs were determined for the antibiotics, and it was noticed that CTX had an MIC of 562 µM for *E. coli* SA/2 and VAN had a MIC of 707 µM for *E. faecalis* B3/101. The results of this assay showed that compounds **2**, **3**, and **7** were able to produce a 2-fold decrease in the MIC of CTX, a third-generation cephalosporin to which *E. coli* SA/2 is resistant, and compound **13** had the same effect on VAN, a glycopeptide with reduced effectivity in *E. faecalis* B3/101.

### 2.4. Efflux Pump Inhibition

Compounds **3**–**13** were assayed using the automated EB method for their ability to modulate ethidium bromide (EB) accumulation on resistant *S. aureus* 272123 and *S.* Typhimurium SL1344 strains. The strains chosen were bacteria relevant both in clinical and food industry settings that have developed not only multidrug resistance via efflux pumps but also other related resistance strategies, such as the formation of biofilms triggered by QS [20]. Therefore, compounds that can circumvent the persistence of these mechanisms are urgently warranted.

*S. aureus* 272123 is a clinical strain, resistant to ofloxacin and methicillin, which has been studied for the expression of the *norA* and *mepA* genes, revealing that the *norA* gene did not change its levels of expression [21]. However, studies have confirmed *norA* to be a core gene of *S. aureus*, being the NorA pump present in all the *S. aureus* strains, even if it is not the main pump responsible for efflux [22].

*S.* Typhimurium SL1344 has the *acrA* gene deleted, which codes for AcrA, the portion with the least predicted affinity. This study aimed to perform an initial screening in the modulation of bacterial efflux pumps of Gram-positive and Gram-negative bacteria, similar to studies previously described [23].

Compounds **3**–**9** and **11**–**13** were tested at the sub-MIC concentration of 50 µM for both bacteria, and compound **10** was tested at one-third of its MIC, 33 µM for *S. aureus* 272123, and 17 µM for *S*. Typhimurium.

Relative fluorescence index (RFI) was calculated based on the means of relative fluorescence units, as can be seen in Table 2. Reserpine and carbonyl cyanide *m*-chlorophenyl hydrazone (CCCP) were used as positive controls for *S. aureus* 272123 and *S*. Typhimurium, respectively, at the sub-MIC concentration of 25 µM.

From analysis of the results, it can be noted that compounds **5** and **6** could increase fluorescence in comparison to the positive control. This can be attributed to the inhibition of the efflux of EB but can also be due to the fluorescence emitted by the compound itself. Analysis of the graphs of the variation of fluorescence throughout the assay (results not shown) suggested that the fluorescence of the compounds was not likely to interfere with the assay. As can be seen, there are compounds, such as **7**, **8**, **9**, **10**, **11**, and **12**, that displayed negative RFI values and, therefore, were considered ineffective for this effect.

**Figure 2:**
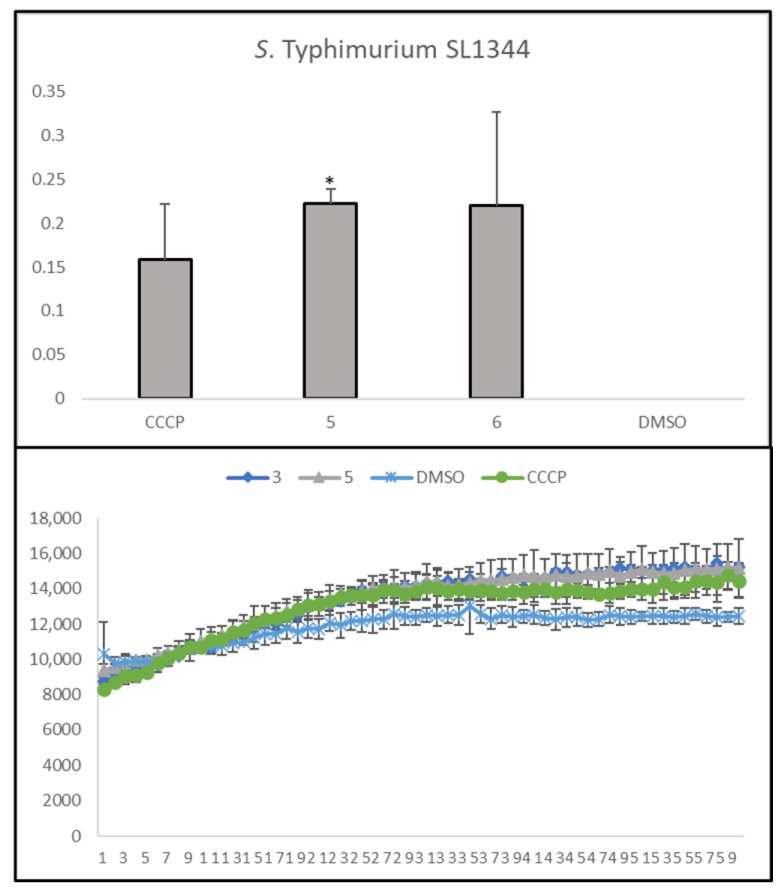
Accumulation of EB in the presence of trimethoxybenzoic acid derivatives **5** and **6** on *S*. Typhimurium SL1344. DMSO: dimethyl sulfoxide (solvent); CCCP: positive control. Statistical comparisons were performed using the *t*-test [* *p* < 0.05 vs. control (DMSO 1% *v*/*v*)].

Compounds **5** and **6** were effective against the efflux of EB in *S*. Typhimurium SL1344 (Figure 2), suggesting that these compounds may present the ability to inhibit bacterial EPs, as EB is a substrate that is transported outside the bacteria by these structures. The compounds tested herein are structurally related to each other, since the only difference between them is the presence of methoxy groups in compounds **5** and **6**, while compounds **9** and **10** have phenolic groups at the same positions. It can be hypothesized that the presence of the phenolic groups can be detrimental to this activity.

**Figure 3:**
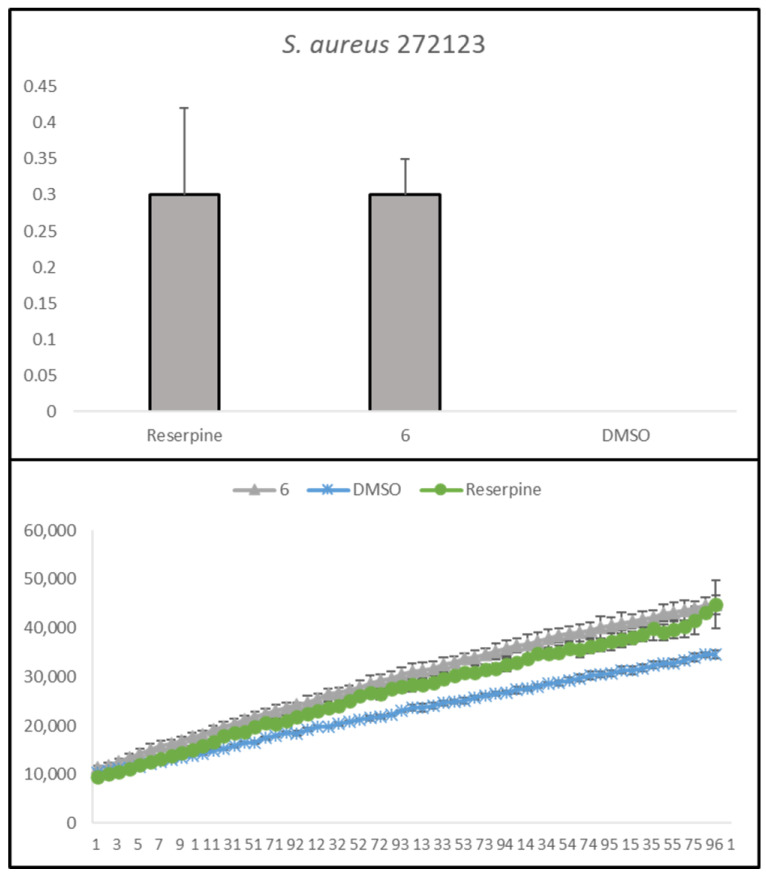
Accumulation of EB in the presence of trimethoxybenzoic acid derivative **6** on *S. aureus* 272123. DMSO: dimethyl sulfoxide (solvent); Reserpine: positive control. Statistical comparisons were performed using the *t*-test [vs. control (DMSO 1% *v*/*v*)].

Compound **6** has the same effect in the MRSA strain studied, as can be seen in Figure 3, where it can be noted that its relative fluorescence units are very close to those of reserpine, used here as a positive control.

Compounds **5** and **6** were visualized in AcrB, as this was the site where they were predicted to bind with the most affinity in the AcrAB-TolC efflux system. A general view of the compounds (Supplementary Material, Figure S5A) shows that compounds **5** and **6**, are predicted to bind similarly within the studied binding site, but to different residues (Supplementary Material).

The results obtained in this assay cannot unequivocally lead to the conclusion that these compounds act as EPIs. There are other mechanisms related to the inhibition of efflux, such as membrane destabilization, or interference with the proton pump or other energy sources. To access the mechanism through which compounds **5** and **6** exert their action, specific studies must be performed to conclude if they act through the direct inhibition of EPs.

### 2.5. Inhibition of Biofilm Formation and Quorum-Sensing Assays

Biofilm formation and QS are related to EPs. The efflux of polymeric substances, used to produce biofilm, can be mediated by EPs. QS signal molecules, which regulate the formation of biofilm, can also be exported by EPs. These factors have an impact on the way bacteria adhere and aggregate on solid surfaces. Additionally, the influence that EPs exert on QS signal molecules can affect QS itself [20].

Due to these relationships, trimethoxybenzoic acid derivatives which had efflux pump activity, **5** and **6**, were evaluated on their effect on biofilm formation by *S. aureus* ATCC 25923 and *S. aureus* 272123. Biofilm inhibition, presented in %, was calculated based on the mean of absorbance units. Reserpine was used as a control in both strains, as this was the positive control used in the real-time EB accumulation assay and has also been described as a biofilm inhibitor [24].

It was shown that compound **5** did not show any influence on biofilm formation, and compound **6** displayed a mild effect on the formation of biofilm in *S. aureus* 272123 (21.3 ± 4.91%), which is still significantly lower than the effect observed with reserpine, herein used as a positive control (72.1 ± 4.24%). Compound **6** was also the only compound tested that could inhibit the efflux of EB in the real-time EB accumulation assay in this strain, which can explain the reduction in biofilm formation. These findings may also corroborate the hypothesis that an efflux system was being inhibited.

In the QS assay, the sensor strain *Chromobacterium violaceum* 026 (CV026) and the acyl-homoserine-lactones (AHL) producer strain *Sphingomonas paucimobilis* Ezf 10–17 (EZF) were inoculated as parallel lines, and the AHL producers *C. violaceum* wild-type 85 (wt85) and *Serratia marcescens* AS-1 were inoculated as a single line. The interaction between the strains and the compounds was evaluated as the reduction of pigment production, in millimeters. The antipsychotic promethazine was used as a positive control [25,26].

Compounds did not display inhibition of QS in these strains, as no discoloration was observed. It has been shown that the species studied in this assay possess efflux systems of the RND family [27], so it could be expected that compounds **5** and **6** would exert some action. However, it is not clear if the results obtained in the real-time EB accumulation assay are connected to the inhibition of EPs of this family.

### 2.6. Antifungal Activity

In an attempt to gain a broader understanding of the antimicrobial activity of the compounds described herein, these synthesized compounds were also evaluated for their antifungal effect in *Candida albicans* ATCC 10231, *Aspergillus fumigatus* ATCC 204305, and *Trichophyton rubrum* FF5. This also adds to the interest that has arisen concerning these compounds as antifouling agents. However, none of them displayed antifungal activity, with the MICs being higher than the maximum concentration tested (128 µg/mL).

### 2.7. In Silico ADME Properties

Structural simplification avoids “molecular obesity” and allows synthetic accessibility but also improves pharmacokinetic profiles and reduces side effects [28]. Some drug-like properties were calculated for the hit compounds **5** and **6** which showed better properties when compared to reserpine, particularly a molecular weight below 500 and a predicted log *P* < 5 (Table 3).

## 3. Materials and Methods

### 3.1. Chemistry

Trimethoxybenzoic acid (**1**, T69000), 4-(2-aminoethyl) morpholine (A55004), and 2-(4-methyl-piperazin-1-yl) ethylamine (CDS003000) were purchased from Sigma-Aldrich (Madrid, Spain), tetrahydrofuran (THF, 348451000), and dichloromethane (CH_2_Cl_2_, 348461000) were purchased from AcrosOrganics (Geel, Belgium), 2-(1*H*-benzotriazole-1-yl)-1,1,3,3-tetramethylaminium tetrafluoroborate (TBTU, B1658) was purchased from TCI (Zwijndrecht, Belgium), and triethylamine (TEA, 489556) from Carlo Erba (Val-de-Reuil, France). TLC separations were performed using Merck silica gel 60 (GF_254_) plates, and flash column chromatography separations were performed using Fluka silica gel 60 (0.04–0.063 mm).

Infrared spectra were recorded in a KBr microplate in a FTIR spectrometer Nicolet iS10 from Thermo Scientific (Waltham, MA, USA) with Smart OMNI—Transmission accessory (Software OMNIC 8.3). ^1^H and ^13^C Nuclear magnetic resonance (NMR) spectra were acquired in CDCl_3_ or DMSO-d_6_ at room temperature on a Bruker Avance 300 (300.13 MHz for ^1^H and 75.47 MHz for ^13^C) or 400 (400.15 MHz for ^1^H and 100.62 MHz for ^13^C) instruments. Chemical shifts were expressed in δ (ppm) values relative to tetramethylsilane (TMS) as an internal reference. High-resolution mass spectrometry (HRMS) was performed on an LTQ OrbitrapTM XL hybrid mass spectrometer (Thermo Fischer Scientific, Bremen, Germany) controlled by LTQ Tune Plus 2.5.5 and Xcalibur 2.1.0. at CEMUP—University of Porto, Portugal.

#### 3.1.1. General Procedure for the Synthesis of Compounds **3**–**13**

The synthesis of compounds **3**–**11** has been previously reported by our group [7] and compound **12** was synthesized accordingly to previous literature [9].

##### 3,4,5-Trimethoxy-*N*-(2-(4-methylpiperazin-1-yl)ethyl)benzamide (**13**)

TBTU (0.568 g, 1.77 mmol) and TEA (82 μL, 0.59 mmol) were added to a solution of compound **1** (0.25 g, 1.18 mmol) in THF (10 mL), at room temperature. After 15 min under stirring, 2-(4-methyl-piperazin-1-yl) ethylamine (0.245 g, 1.71 mmol) was added. The reaction was left stirring overnight. The solvent was evaporated to dryness and the residue was dissolved in ethyl acetate, extracted twice with a saturated solution of NaHCO_3_, and washed twice with water. The organic phase was dried over anhydrous sodium sulphate. The residue was further purified by crystallization with ethyl acetate to afford white crystals (0.14 g, 0.43 mmol, 36% yield).

IR (KBr) ʋ_max_: 3432, 3300, 2936, 2812, 2789, 1633, 1582, 1541, 1505, 1460, 1413, 1345, 1235, 1130, 992, 844, 733 cm^−1^; ^1^H NMR (CDCl_3_, 400.14 MHz) *δ*: 7.01 (2H, s, H-2′,6′), 6.80 (1H, brt, NH amide), 3.91 (6H, s, 3′,5′-OCH_3_), 3.88 (3H, s,4′- OCH_3_), 3.54–3.50 (2H, m, CH_2_CH_2_), 2.63–2.60 (2H, m, CH_2_CH_2_), 2.55–2.45 (8H, m, H-piperazine), 2.28 (3H, s, piperazine-CH_3_) ppm (Figure S1); ^13^C NMR (CDCl_3_, 100.62 MHz) *δ*: 167.1 (C=O amide), 153.3 (C-3′,5′), 141.0 (C-4′), 130.3 (C-1′), 104.5 (C-2′,6′), 61.1 (4′-OCH_3_), 56.4 (3′,5′-OCH_3_), 56.3 (C-piperazine), 55.5 (C-piperazine), 52.9 (CH_2_CH_2_), 46.2 (CH_3_-piperazine), 36.5 (CH_2_CH_2_) ppm (Figure S2); HRMS (ESI-) m/z: [M-H]^−^ calcd. for C_17_H_26_N_3_O_4_ 336.19292, found 336.19415 (Figure S3).

### 3.2. Molecular Docking

The crystal structure of the AcrB (PDB: 2DRD) [29], AcrA (PDB: 2F1M) [30], and TolC (PDB: 1EK9) [31] portions of the AcrAB-TolC bacterial efflux system, downloaded from the protein databank (PDB) [32], were used for this study. The known inhibitor reserpine, along with the tested compounds were drawn with ChemDraw (PerkinElmer Informatics) and minimized using ArgusLab. Docking was carried out using AutoDock Vina (Scripps, CA, USA) [33], in the sites described in [11,16]. The NorA efflux pump does not have an available crystal structure, and a homology model was prepared. The model was generated using the Swiss Model server [34] and the sequence deposited in Uniprot (Q5HHX4) [35], using the EmrD pump from *E. coli* (PDB: 2GFP) as the homolog, as described in [36]. The sequence similarity was 0.28, the coverage was 0.91 and the sequence identity 17.33%. The site analyzed was that described in [10]. The top nine poses were collected for each molecule and the lowest docking score value was associated with the most favorable binding conformation.

### 3.3. Culture Media and Chemicals

The culture media used in the experiments were the following: cation-adjusted Mueller-Hinton broth (MHB II; Sigma-Aldrich, St- Louis, MO, USA and Biokar Diagnostics, Allone, Beauvais, France), Luria-Bertani broth (LB-B; Sigma, St. Louis, MO, USA), Tryptic Soy broth (TSB; Scharlau Chemie S. A., Barcelona), and Trypto-Casein Soy agar (TSA; Biokar Diagnostics, Allone, Beauvais, France). Modified Luria-Bertani agar (LB*-A), used for the QS inhibition assays, was prepared in-house, according to the formula: 1.0 g yeast extract (Merck, Darmstadt, Germany), 10.0 g tryptone (Biolab, Budapest, Hungary), 10.0 g NaCl (Molar Chemicals, Halásztelek, Hungary), 1.0 g K_2_HPO_4_ (Biolab, Budapest, Hungary), 0.3 g MgSO_4_.7H_2_O (Reanal, Budapest, Hungary), 5 mL Fe-EDTA stock solution and 20.0 g of bacteriological agar (Molar Chemicals, Halásztelek, Hungary) per 1 L of media. Sabouraud Dextrose Agar (SDA) from bio-Mérieux (Marcy L’Etoile, France), RPMI-1640 broth medium (containing L-glutamine and the pH indicator phenol red but without bicarbonate) from Biochrom AG (Berlin, Germany) buffered with 3-(*N*-morpholino) propanesulfonic acid (MOPS) from Sigma-Aldrich (St. Louis, MO, USA) to pH 7.0 were used for antifungal activity evaluation.

Dimethyl sulfoxide (DMSO), phosphate-buffered saline (PBS; pH 7.4), EB, reserpine, CCCP, promethazine, ciprofloxacin, and crystal violet (CV) were purchased from Sigma-Aldrich Chemie GmbH (Steinheim, Germany). The antibiotic CTX was purchased from Duchefa Biochemie (Haarlem, The Netherlands), and VAN from Oxoid (Basingstoke, England).

### 3.4. Microorganisms

As Gram-positive strains, *S. aureus* ATCC 25923, *E. faecalis* ATCC 29212, methicillin and ofloxacin-resistant *S. aureus* 272123 clinical isolate, and VRE *E. faecalis* B3/101 [37] were used. As Gram-negative strains, *E. coli* ATCC 25922, *P. aeruginosa* ATCC 27853, the *acrA* gene inactivated mutant *S. enterica* serovar Typhimurium SL1344, and clinical isolates of the ESBL *E. coli* SA/2 were investigated in this study.

For the QS tests, all the strains used were Gram-negative. The bacteria used were *C. violaceum* wild type 85 (wt85) characterized by the AHL signal molecule-mediated production of the purple violacein pigment, capable of endogenous QS-signal molecule-production (*N*-hexanoyl-l-HSL), *C. violaceum* CV026 (CV026), a Tn5 transposase-mutant, AHL-signal molecule indicator strain (produces purple violacein pigment in the presence of AHL), which is incapable of endogenous QS-signal molecule-production, but useful in the detection of external stimuli, and *S. paucimobilis* Ezf 10–17 (EZF), an AHL-producing-strain (used with *C. violaceum* CV026), and *S. marcescens* AS-1, characterized by the production AHL signal molecule-mediated production of the orange-red pigment prodigiosin (2-methyl-3-pentyl-6-methoxyprodigiosin), capable of endogenous QS-signal molecule-production (*N*-hexanoyl-l-HSL) [38].

As for fungi, the strains used were *Candida albicans* ATCC 10231, *Candida krusei* ATCC 6258, *Aspergillus fumigatus* ATCC 204305, and a clinical strain of dermatophyte *Trichophyton rubrum* FF5.

### 3.5. Antibacterial and Antibiotic Potentiation Assays

Antibacterial activity was assessed by the determination of the MIC of the synthesized compounds by visual inspection. The microdilution method, in a 96-well plate, according to the Clinical and Laboratory Standard Institute (CLSI) guidelines [39] was used. The media used was MHB II. The concentrations tested ranged from 100 µM to 0.195 µM and were prepared from a stock solution of 10 mM in DMSO. DMSO was used in subinhibitory concentrations (1% *v*/*v*). Ciprofloxacin was used as a positive control for all the strains tested. The combined effect of compounds **3**–**13** and clinically relevant antimicrobial drugs were evaluated by determining the antibiotic’s MIC in the presence of each compound. Briefly, the MIC values of CTX and VAN, for *E. coli* SA/2 and *E. faecalis* B3/101, respectively, were determined in the presence of the highest concentration of each compound tested in previous assays where there was bacterial growth. The antibiotic tested was serially diluted whereas the concentration of each compound was kept fixed. Antibiotic MICs were determined as described above.

### 3.6. Efflux Pump Inhibition

The synthesized compounds **5**–**13** were evaluated for their ability to inhibit efflux pumps in *S*. Typhimurium SL1344 and *S. aureus* 272123 using real-time fluorimetry, monitoring the intracellular accumulation of EB, an efflux pump substrate. This was determined by the automated method using a CLARIOstar Plus plate reader (BMG Labtech, Ortenberg, Germany). Reserpine and CCCP were applied at 25 µM as positive controls, and the solvent DMSO was applied at 1% (*v*/*v*). The bacterial strains were incubated in an appropriate culture media (TSB—*S. aureus* 272123; LB-B—*S*. Typhimurium SL1344) at 37 °C until they reached an optical density (OD) of 0.6 at 600 nm. The culture was centrifuged at 13,000× *g* for 3 min, and the pellet was washed and resuspended with phosphate buffered saline (PBS, pH 7.4). The suspension was centrifuged again in the same conditions and resuspended in PBS. The compounds were applied at 50 µM in a solution of a non-toxic concentration of EB (1 µg/mL) in PBS, except for compound **10**, which was applied at 17 µM against *S.* Typhimurium SL1344 and 33 µM against *S. aureus* 272123 (one-third of the MIC) Then, 50 µL of this solution were transferred into a 96-well black microtiter plate (Greiner Bio-One Hungary Kft, Hungary), and 50 µL of bacterial suspension (OD_600_ 0.4–0.6) were added to each well. The plates were placed into the CLARIOstar plate reader, and the fluorescence was monitored at excitation and emission wavelengths of 530 nm and 600 nm, respectively, every minute for one hour on a real-time basis. From the real-time data, the activity of the compounds, namely the relative fluorescence index (RFI) of the last time point (minute 60) of the EB accumulation assay, was calculated according to the following formula:

$$\mathrm{RFI}=\frac{{\mathrm{RF}}_{\mathrm{treated}}-{\mathrm{RF}}_{\mathrm{untreated}}}{{\mathrm{RF}}_{\mathrm{untreated}}}$$

where RF_treated_ is the relative fluorescence (RF) at the last time point of the EB accumulation curve in the presence of the compound, and RF_untreated_ is the RF at the last time point of the EB accumulation curve of the untreated control, having only the solvent (DMSO) control [40]. The accumulation curves were designed using Microsoft Excel^®^. The samples were tested in triplicate, and the RFI presented results from the average of these three values. The accumulation curves show the mean of the RF over the cycles performed. Standard deviation (SD) was calculated automatically and included in the RF curves.

### 3.7. Inhibition of Biofilm Formation

Compounds **5** and **6**, and **10** were tested for their ability to decrease the formation of biofilm. The strains used were the Gram-positive *S. aureus* ATCC 25923 and *S. aureus* MRSA 272123. The detection of biofilm formation was possible with the use of the dye, CV (0.1% *v*/*v*). The initial inoculum was incubated in TSB overnight and then diluted to an OD_600_ of 0.1. Then, the bacterial suspension was added to 96-well microtiter plates and compounds **5** and **6** were added at 100 µM for both strains, while compound **10** was added at 100 μM for *S. aureus* ATCC 25923 and 50 µM in *S. aureus* 272123. The final volume in each well was 200 µL. Reserpine was used as a positive control. The plates were incubated at 30 °C for 48 h with gentle stirring (100 rpm). After this incubation period, the TSB medium was discarded and the plates were washed with tap water to remove unattached cells. Afterward, 200 µL of a 0.1% (*v*/*v*) CV solution was added to the wells and incubated for 15 min at room temperature. Then, the CV solution was removed from the wells, the plates were washed again with tap water, and 200 µL of a 70% ethanolic solution was added to the wells. Biofilm formation was determined by measuring the OD_600_ using a Multiscan EX ELISA plate reader (Thermo Labsystems, Cheshire, WA, USA). The anti-biofilm effect of the compounds was expressed in the percentage (%) of decrease in biofilm formation [41].

### 3.8. Quorum-Sensing Inhibition

The QS inhibitory effect of the compounds was examined on the EZF and the sensor CV026 strains, on the wt85 strain, and on *S. marcescens*, for the trimethoxybenzoic acid derivatives **5**, **6**, and **10**. The parallel inoculation method was used, where pair combinations of the used sensor strain CV026 and the AHL-producing strain EZF were inoculated directly onto the LB*-A agar surface in parallel at an approximate distance of 5 mm from each other. *S. marcescens* AS-1 and wt85 were inoculated as a single line. Filter paper disks (7 mm in diameter) were placed on the center of the inoculated line(s) and impregnated with 8 µL of a solution of 10 mM of the compounds. Promethazine was used as the positive control. The agar plates were incubated at room temperature (20 °C) for 24–48 h. The QS inhibition was accessed visually, through the inhibition of pigment production. The discolored, but intact, bacterial colonies were measured with a ruler [26].

### 3.9. Antifungal Assays

A dilution series (1:2) of the compounds dissolved in DMSO was prepared in the RPMI medium, with concentrations ranging between 256 and 8 µg/mL.

Susceptibility tests were performed by the broth microdilution method based on the Clinical and Laboratory Standards Institute (CLSI); M27A-3 for yeasts (*C. albicans*) and M38-A2 [42] for filamentous fungi (*A. fumigatus* and *T. rubrum*) [43].

Briefly, from yeast colonies growing on SDA (24 h incubation) a suspension with 10^3^ cells/mL was prepared in RPMI-1640. For filamentous fungi, well-sporulated cultures on SDA were used to obtain a final suspension of spores in RPMI (0.4–5 × 10^4^ spores/mL, for *A. fumigatus*, and 1–3 × 10^3^ spores/mL, for the dermatophyte). Using 96-well plates, equal amounts (100 µL) of cells/spores suspension and compound dilution were mixed and incubated for 48 h at 35 °C for *C. albicans* and *A. fumigatus* and 5–7 days at 25 °C for *T. rubrum*. To confirm microorganism viability, the medium sterility, and the effect of DMSO used, a positive control (microorganism in culture medium) representing 100% growth, a negative control (culture medium) corresponding to 0% growth, and a DMSO control (microorganism in culture medium with DMSO 2.0% *v*/*v*) were included. Quality control was ensured by the assessment of the activity of a commercial antifungal (voriconazole) with a reference yeast for quality control and recommended by CLSI, *C. krusei* ATCC 6258. The interpretation of the results was based on the presence of turbidity and MIC is defined as the lowest concentration that totally inhibits growth when compared to positive control.

## 4. Conclusions

Docking studies targeting portions of the AcrAB-TolC bacterial efflux system and the NorA efflux pump (EP) were performed on an in-house library of trimethoxybenzoic acid and gallic acid derivatives. A new amide derivative (**13**) was synthesized for the first time in a one-step procedure in moderate yields.

Among the investigated compounds, derivatives **5** and **6** showed the ability to inhibit efflux pumps in the *acrA* gene-inactivated mutant *S. enterica* serovar Typhimurium SL1344, and **6** also inhibited EPs in *S. aureus* 272123. Genetic assays could be performed to assess which EP was being inhibited by these two compounds. Only compound **10** showed antibacterial activity against the two bacterial strains studied. Derivatives **5**, **6**, and **10** did not show any activity in both the biofilm and quorum sensing assays.

Structure-activity relationship (SAR) studies showed that better activity was obtained when the phenyl ring was substituted with three methoxy groups. The presence of an aromatic moiety linked by an aliphatic chain to the trimethoxybenzoic acid could also be essential to the bioactivity, as well as two secondary amines, as the compounds predicted to have better docking scores were the ones that possess these features.

The feasible synthesis and their molecular weight below 500 and predicted log *p* values < 5 make these compounds potential candidates as EP inhibitors in multidrug efflux systems, although further studies may be conducted to assess this potential.

Overall, the potential of amide trimethoxybenzoic acid derivatives as efflux pump inhibitors to combat antibiotic resistance in multidrug-resistant pathogenic bacteria such as MRSA was disclosed.

## Figures and Tables

**Table 1:** Docking results for compounds **1–13** in the different portions of the AcrAB-TolC efflux system and the homology model of NorA.

Compound	Docking Score				
	AcrB	AcrA		TolC	NorA
		HH	LD		
**1**	−5.9	−5.1	−4.5	−5.5	−4.1
**2**	−5.6	−4.3	−3.7	−5.5	−4.0
**3**	−6.5	−4.9	−4.3	−6.0	−4.5
**4**	−6.2	−5.5	−4.6	−5.9	−4.2
**5**	−7.3	−5.7	−4.7	−6.4	−7.3
**6**	−7.6	−6.4	−5.4	−6.7	−6.2
**7**	−6.8	−5.0	−6.0	−5.7	−4.4
**8**	−6.7	−5.0	−5.8	−5.9	−4.7
**9**	−8.9	−5.8	−6.7	−6.5	−5.9
**10**	−9.8	−6.5	−7.1	−7.3	−6.2
**11**	−6.6	−4.7	−5.3	−5.7	−3.9
**12**	−7.0	−5.0	−5.0	−5.9	−4.8
**13**	−7.1	−5.0	−5.6	−5.9	−4.8
Reserpine	−8.1	5.6	4.6	−7.5	−4.1

**HH**: Helical hairpin; **LD**: Lipoyl domain.

**Table 2:** RFI of tested derivatives.

Compound	RFI ± SD	
	*S. aureus* 272123	*S.* Typhimurium SL1344
**3**	ND	ND
**4**	ND	ND
**5**	0.08 ± 0.07	0.22 ± 0.02
**6**	0.30 ± 0.05	0.22 ± 0.11
**7**	−0.02 ± 0.01	−0.07 ± 0.03
**8**	−0.07 ± 0.03	−0.07 ± 0.05
**9**	−0.35 ± 0.01	−0.41 ± 0.01
**10**	−0.21 ± 0.03	−0.36 ± 0.01
**11**	−0.03 ± 0.03	−0.06 ± 0.01
**12**	0.17 ± 0.03	−0.01 ± 0.01
**13**	0.14 ± 0.02	0.06 ± 0.01
Reserpine	0.30 ± 0.12	0.04 ± 0.07
CCCP	ND	0.16 ± 0.06

SD—standard deviation; ND—not determined; CCCP—carbonyl cyanide *m*-chlorophenyl hydrazone.

**Table 3:** Swiss ADME parameters for Lipinski’s rules for reserpine, compound **5** and compound **6**.

Compound	Molecular Weight	# Rotatable Bonds	# H-Bond Acceptors	# H-Bond Donors	ilogP
Reserpine	608.68	10	10	1	5.16
**5**	330.38	9	4	2	3.12
**6**	355.39	8	5	2	2.46

## Data Availability

Not applicable.

## Supplementary Materials

The following supporting information can be downloaded at: https://www.mdpi.com/article/10.3390/ijms232214468/s1.
