# Supplementary material for: Derivatives of Trimethoxybenzoic Acid and Gallic Acid as Potential Efflux Pump Inhibitors: In Silico and In Vitro Studies
Source: Int J Mol Sci. 2022 Nov 21;23(22):14468. doi: 10.3390/ijms232214468 (PMC9699367; doi:10.3390/ijms232214468)
Supplement: Supplementary file 1 [file ijms-23-14468-s001.zip › ijms-2014807-supplementary.pdf]

International Journal of Molecular Sciences

Supplementary Materials

Derivatives of trimethoxybenzoic acid and gallic acid as potential efflux pump inhibitors: *in silico* and *in vitro* studies

Ana Rita Neves [1,2] †, Fernando Durães [1,2] †, Joana Freitas-Silva [2,3], Nikoletta Szemerédi [4], Paulo Martins-da-Costa [2,3], Eugénia Pinto [2,5], Marta Correia-da-Silva [1,2*], Gabriella Spengler [4], and Emília Sousa [1,2*]

1 Laboratory of Organic and Pharmaceutical Chemistry, Faculty of Pharmacy, University of Porto, Porto, Portugal
2 Interdisciplinary Centre of Marine and Environmental Research (CIIMAR), University of Porto, Portugal
3 ICBAS – Institute of Biomedical Sciences Abel Salazar, University of Porto, Rua de Jorge Viterbo Ferreira 228, 4050-313 Porto, Portugal
4 Department of Medical Microbiology, Albert Szent-Györgyi Health Center and Albert Szent-Györgyi Medical School, University of Szeged, Semmelweis utca 6, 6725 Szeged, Hungary
5 Laboratory of Microbiology, Department of Biological Sciences, Faculty of Pharmacy, University of Porto, Rua de Jorge Viterbo Ferreira 228, 4050-313 Porto, Portugal
* Correspondence: esousa@ff.up.pt (E.S.); m_correiadasilva@ff.up.pt (M.C.S.).
† These authors contributed equally to this work and share first authorship.

International Journal of
Molecular Sciences

MDPI

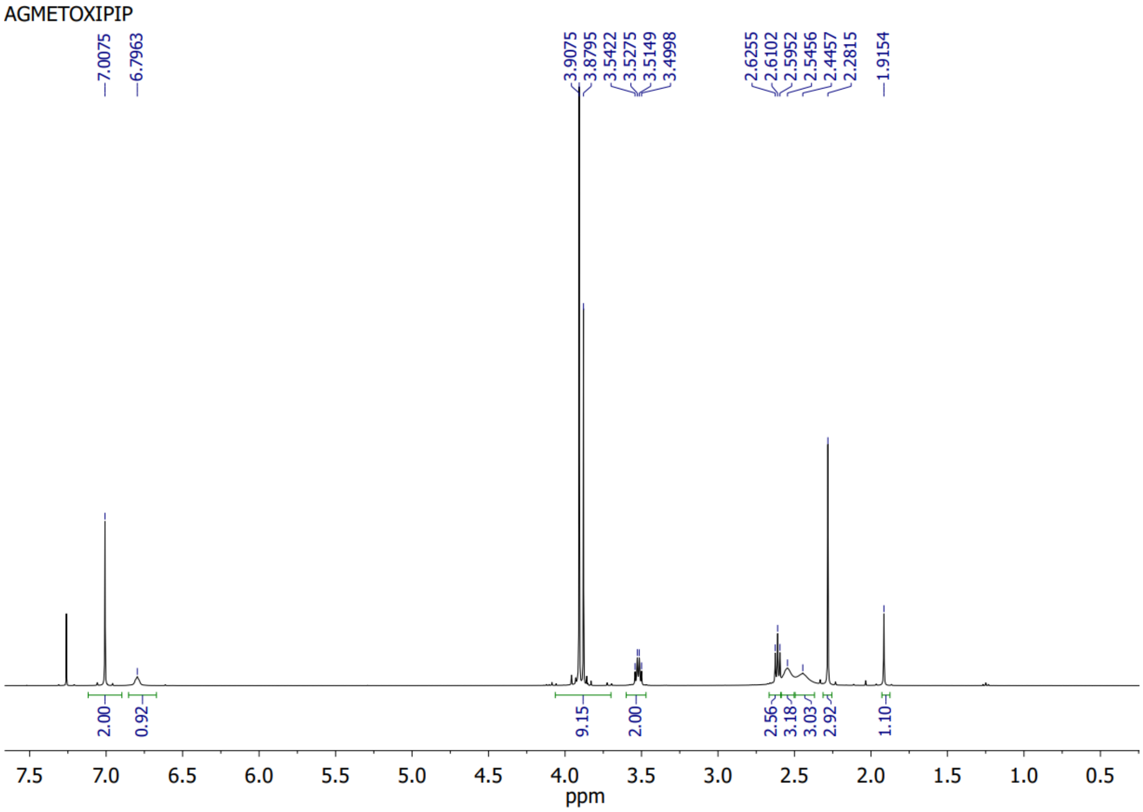

Figure S1 ^{1}H NMR ($CDCl_3$, 400.14 MHz) spectrum of compound **13**.

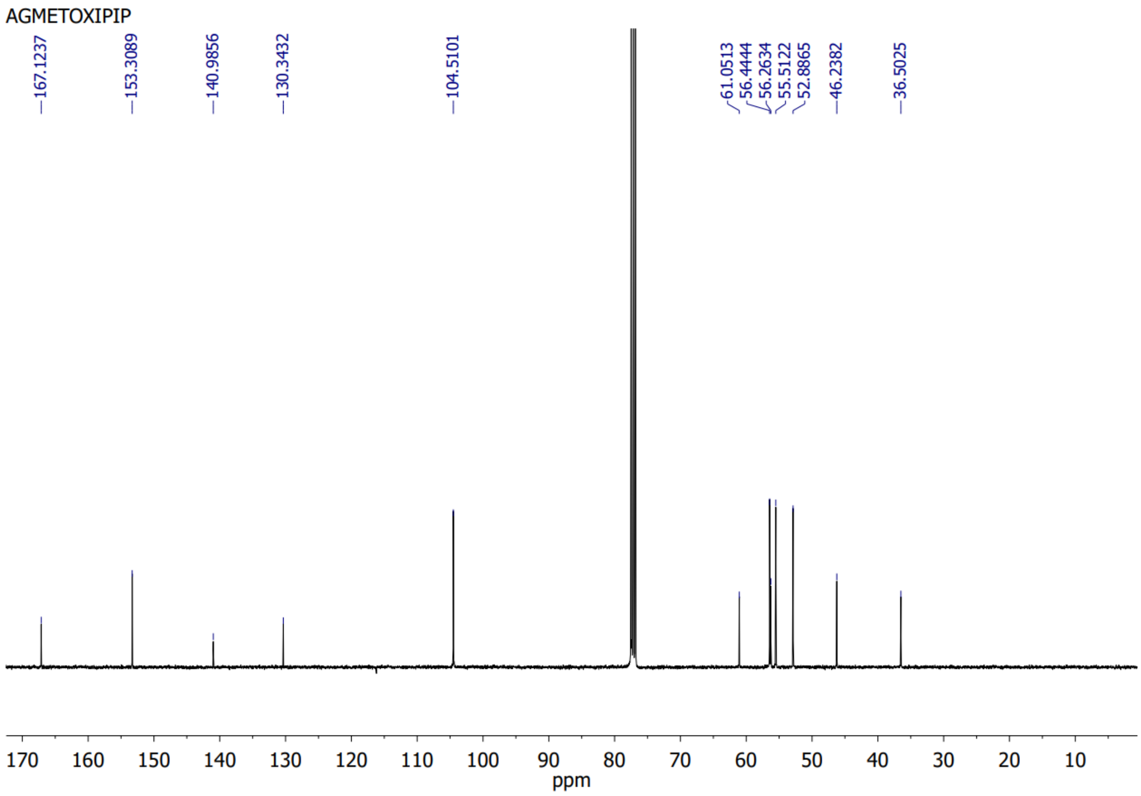

Figure S2 ^{13}C NMR ($CDCl_3$, 100.62 MHz) spectrum of compound **13**.

International Journal of Molecular Sciences

MDPI

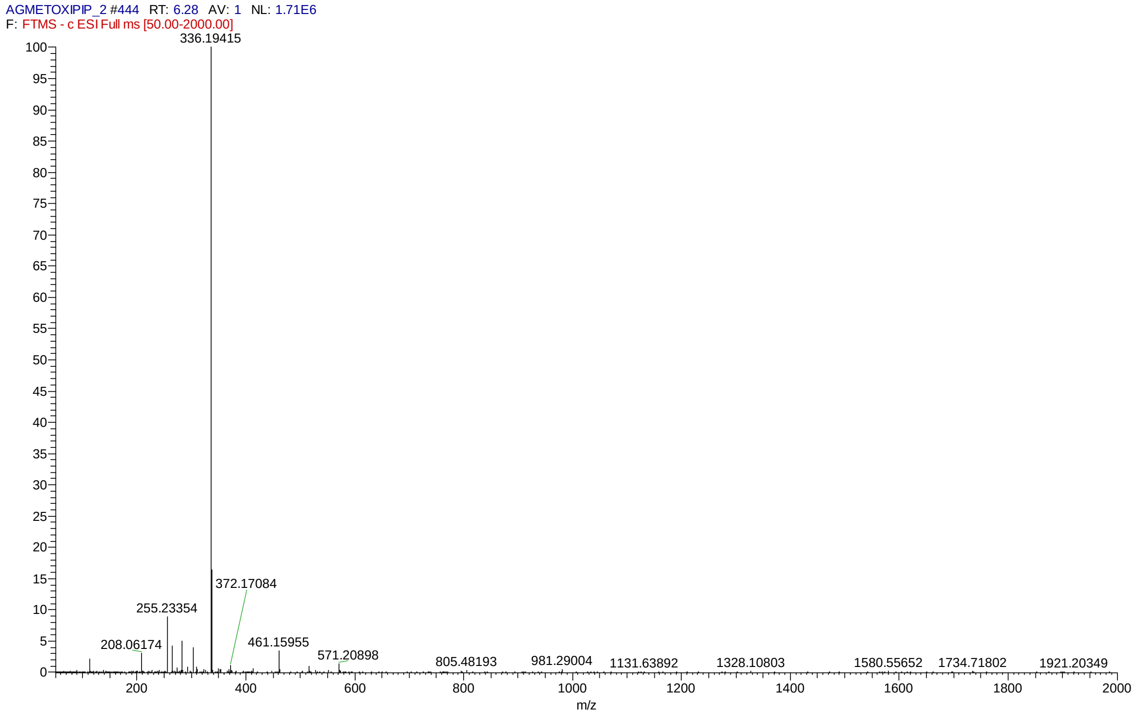

Meas. m/z	Formula	m/z	err [ppm]
336.19292	$C_{17}H_{26}N_3O_4$	336.19415	3.65

Figure S3 Electrospray ESI data for compound **13**.

Molecular visualization

The compounds were visualized using PyMOL, in the site that had previously been described for reserpine in AcrB and NorA. According to the docking scores obtained in **Table 1**, the compounds chosen for visualization were **5**, **6**, **9**, and **10** in both models, as these were the ones with the best docking scores. **Figure S4** illustrates these results.

Concerning AcrB, what is immediately noticed is that fact that the trimethoxybenzoic acid moieties of the tested compounds and reserpine do not overlap and are even in different regions within the defined site (**Figure S4A**). Starting with the analysis of reserpine, it can be noticed that the trimethoxybenzoic acid moiety is predicted to be responsible for the interactions between the ligand and the target protein. In fact, a hydrogen bond is predicted to happen between one of the methoxyls and Lys-163. Further interactions noticed were a polar interaction between the carbonyl and Leu-113, and between the ester oxygen and Ser-128 (**Figure S4B**).

Compounds **5** and **6** were predicted to bind in the same site and interact with the same residues. Two hydrogens interactions were predicted to be established: one between one of the methoxyl groups and Phe-136, and another between the nitrogen in the amide moiety and Gln-176 (**Figure S4C**). The corresponding hydroxylated derivatives **9** and **10** had a binding site close to **5** and **6**, with a hydrogen bond predicted to happen between

one of the hydroxyls and Gln-176. Furthermore, the hydroxyls are also predicted to establish hydrogen interactions with Ile-291 and Lys-292 (**Figure S5D**).

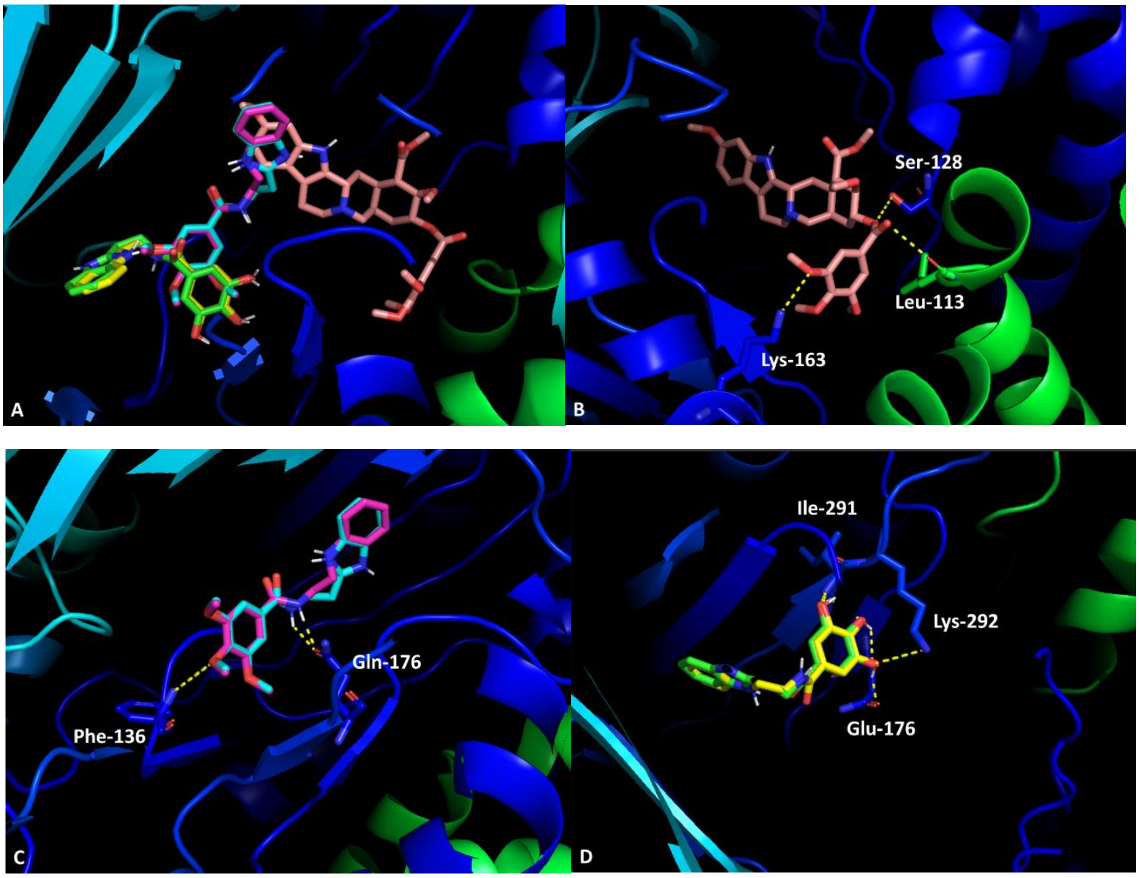

Figure S4 (A) Visualization of reserpine (light pink) and compounds **5** (pink), **6** (blue), **9** (yellow), and **10** (green) in the reserpine binding site of AcrB; (B) Predicted interactions between compounds **5** and **6** and AcrB; (C) Predicted interactions between compounds **9** and **10** and AcrB.

In the case of NorA (**Figure S5**), the methoxybenzoic/phenolic moieties in the compounds are predicted to bind very close to the trimethoxybenzoic moiety in reserpine (**Figure S5A**). Reserpine is predicted to establish two polar interactions with the carboxylic acid in Asp-352 (**Figure 5B**).

The trimethoxy derivatives **5** and **6** are predicted to establish different interactions. Compound **5** is likely to establish a hydrogen bond with Ser-279, whereas **6** is predicted to establish a polar interaction between the carbonyl and Asp-352 (**Figure S5C**). Compound **9** did not show any predicted hydrogen interaction, but other kinds of interactions not shown with this method are still likely to occur, such as van der Waals. Compound **10**, on the other hand, showed a hydrogen interaction between a hydroxyl and Ile-346 (**Figure S5D**). It should, however, be noted that in a homology model there is a greater chance of uncertainty.

International Journal of
Molecular Sciences

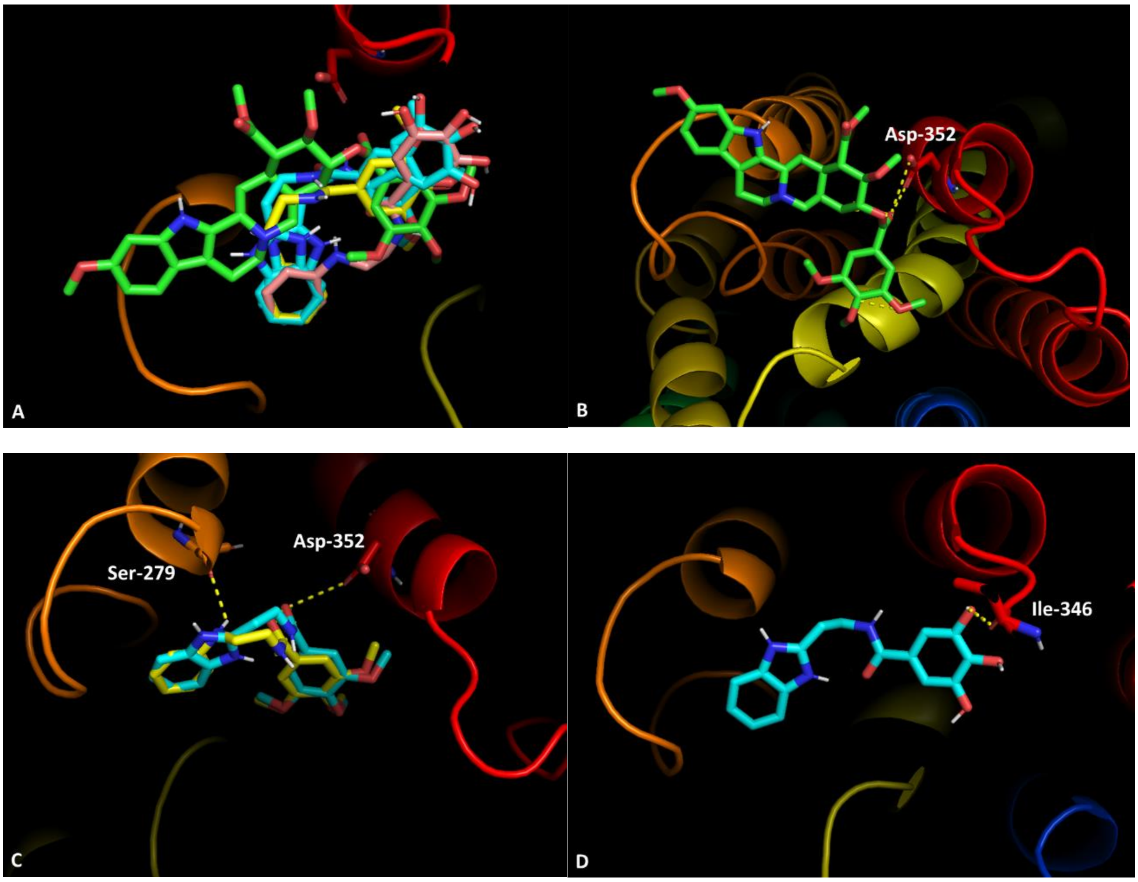

Figure S5 (A) Visualization of reserpine (green) and compounds **5** (yellow), **6** (blue), **9** (pink), and **10** (blue) in the reserpine binding site of NorA; (B) Predicted interactions between compounds **5** and **6** and NorA; (C) Predicted interactions between compound **10** and NorA.
